# The blood labyrinthine barrier in the human normal and Meniere’s disease macula utricle

**DOI:** 10.1038/s41598-017-00330-5

**Published:** 2017-03-21

**Authors:** Gail Ishiyama, Ivan A. Lopez, Paul Ishiyama, Harry V. Vinters, Akira Ishiyama

**Affiliations:** 1 Department of Neurology, David Geffen School of Medicine, University of California, Los Angeles, USA; 2 Department of Head and Neck Surgery, David Geffen School of Medicine, University of California, Los Angeles, USA; 3 Department of Pathology & Laboratory Medicine (Neuropathology), David Geffen School of Medicine, University of California, Los Angeles, USA

## Abstract

The ultrastructural organization of the blood labyrinthine barrier (BLB) was investigated in the human vestibular endorgan, the utricular macula, using postmortem specimens from individuals with documented normal auditory and vestibular function and surgical specimens from patients with intractable Meniere’s disease. Transmission electron microscopic analysis of capillaries located in the normal human utricular stroma showed vascular endothelial cells with few pinocytotic vesicles, covered by a smooth and uniform basement membrane surrounded by pericyte processes. Meniere’s disease specimens revealed differential ultrastructural pathological changes in the cellular elements of the microvasculature. With moderate degeneration of the BLB, there were numerous vesicles within the vascular endothelial cells (VECs), with increased numbers at the abluminal face, pericyte process detachment and disruption of the perivascular basement membrane surrounding the VECs. With severe degeneration of the BLB, there was severe vacuolization or frank apparent necrosis of VECs and loss of subcellular organelles. A higher severity of BLB degenerative changes was associated with a higher degree of basement membrane thickening and edematous changes within the vestibular stroma. This study presents the first ultrastructural analysis of the capillaries constituting the BLB in the human vestibular macula utricle from normal and Meniere’s disease.

## Introduction

In the inner ear, the term blood labyrinthine barrier (BLB) refers to the barrier between the vasculature and the inner ear fluids, either endolymph or perilymph^1, 2^. The BLB is critical for the maintenance of the inner ear fluid ionic homeostasis and for the prevention of the entry of deleterious substances into the inner ear^3^. By studying the penetration of dyes and drugs from the systemic circulation into the fluids of the inner ear, it can be shown that the BLB is selective and that the composition of the inner ear fluids is regulated and markedly different from that of blood or of other fluids such as cerebrospinal fluid^2^.

Understanding the dynamics of the BLB is important to develop therapeutic drug delivery systems to the inner ear to block or enhance the BLB inflammatory response. The BLB in the cochlea is well studied in animal models^2, 4, 5^. Initial transmission electron microscopy (TEM) studies demonstrated that the BLB in the guinea pig stria vascularis and spiral ligament is composed of vascular endothelial cells (VECs) surrounded by a basement membrane^6^. More recent studies have noted that, in addition, the intrastrial BLB is composed of pericytes and perivascular-resident macrophage-like melanocytes^7–11^. The VECs line the interior surface of the blood vessels, connected by tight junctions, forming an interface between circulating blood and the rest of the vessel wall. Pericytes are the only cell type to intimately connect with VECs as they lie embedded within the endothelial basement membrane. The perivascular-resident macrophage-like melanocytes have foot processes on the outer surface of capillaries and these cells may play a similar role as astrocytes in the brain to maintain the integrity of the barrier and may play a role in inflammatory responses^3, 12^. The BLB within the perilymph-filled areas that are part of the blood-perilymph barrier include the cochlear spiral ligament, spiral limbus, modiolus, osseous spiral lamina, vestibular stromal vasculature and the sub-epithelial dark cell area^13^. The vestibular BLB unit in the rodent is similar to the rodent strial BLB unit at the light microscopic level: composed of vascular endothelial cells, pericytes and perivascular-resident macrophage-like melanocytes^10^.

Recent studies have implicated loss of the integrity of the BLB in several inner ear pathologies including acoustic trauma, autoimmune inner ear disease, and presbycusis^3, 7, 8, 14–16^. Meniere’s disease is a disabling syndrome of fluctuating hearing loss, episodic vertigo, and ear fullness, the etiology and pathophysiology of which remain poorly understood. Endolymphatic hydrops, a ballooning of the endolymph fluid, was demonstrated in postmortem human temporal bone studies on patients with Meniere’s disease^17, 18^. Endolymphatic hydrops is characterized by an excess of endolymph; however, the mechanism whereby vertigo and hearing loss occur is unclear^19–21^. MRI studies have demonstrated that within patients with Meniere’s disease, the degree of hydrops correlates with the degree of hearing loss^22^. Hypothesized mechanisms for hearing loss in Meniere’s disease include a decrease in cochlear blood flow to one-third as a direct effect of hydrops^23^; however, cochlear blood flow measurement in subjects with Meniere’s disease did not differ from control groups^24^. While pathophysiology of the BLB has been proposed in Meniere’s disease, no prior studies have evaluated the BLB ultrastructure in normal humans or in Meniere’s disease. Of note, Meniere’s disease is a uniquely human disease and there are no animal models which replicate the disease.

The normal vascular anatomy of the cochlea has been well documented using light, TEM and scanning EM of corrosion cast of the cochlear vasculature^25^. To our knowledge, there are no previous ultrastructural studies of the human vestibular stromal capillaries. We have previously documented pathological alterations of the vestibular crista ampullaris sub-epithelial basement membrane in patients with intractable Meniere’s disease^26^. The utricular stroma demonstrated thickening of the basement membrane, fibroblast vacuolization and fibrillary deposition close to the blood vessels. However, the ultrastructural morphology of the human vestibular BLB has not yet been investigated. We report the first ultrastructural study of the normal human BLB of capillaries located underneath the utricular sensory epithelia and compare the results with those from Meniere’s patients.

## Results

### The normal human BLB in the capillaries of the utricle stroma

**Figure 1:**
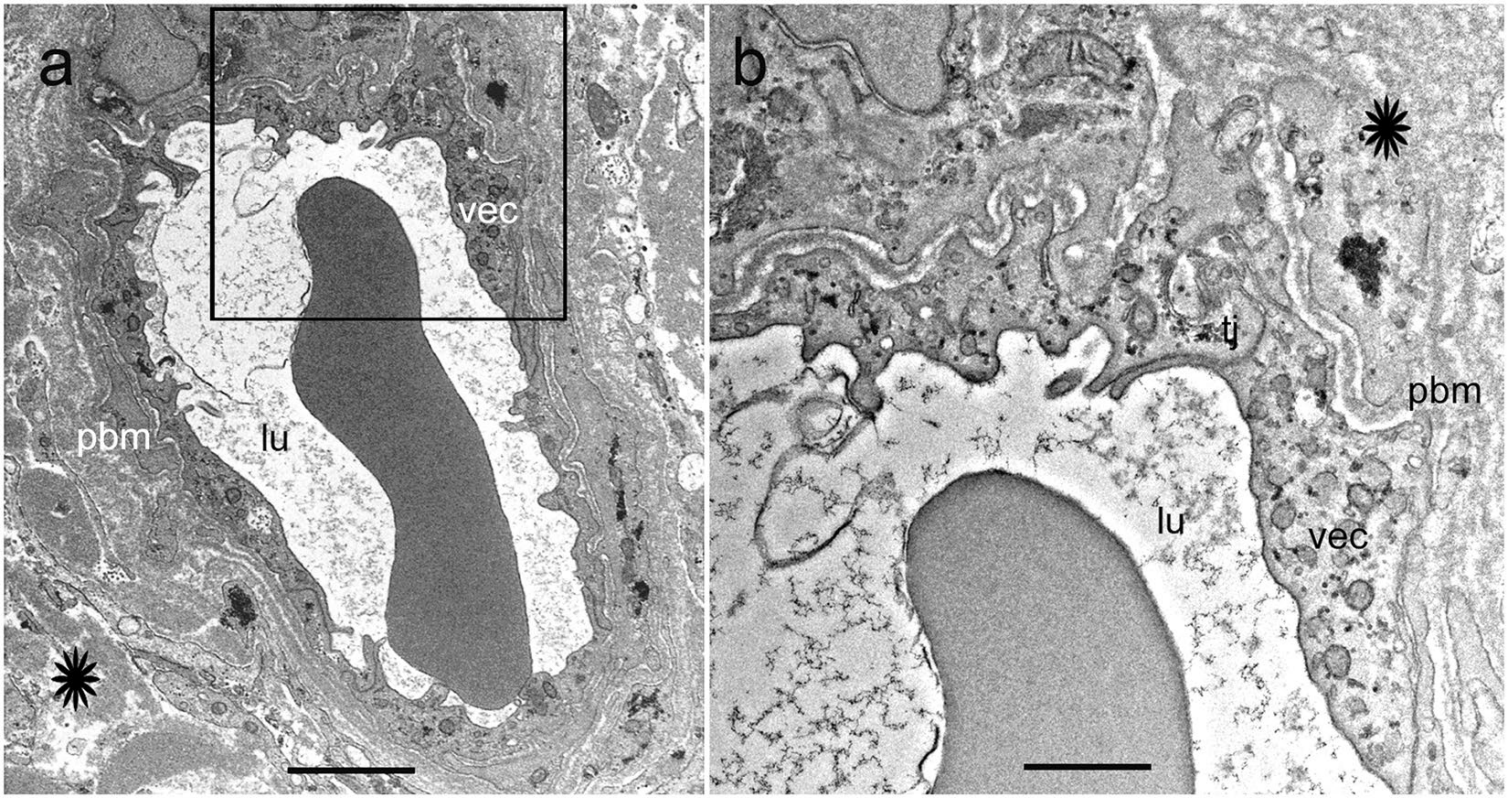
The BLB in normal capillaries. Capillary located in the stroma of the macula utricle from a normal subject (62-year-old male). (**a**) Low magnification view. The lumen (lu) of the capillary is smooth and vascular endothelial cells (vec) exhibit normal morphology. The perivascular  basement membrane (pbm) and extracellular matrix (asterisk) are also normal. (**b**) high magnification view from (**a**), vec show normal cytoplasm. Tight junctions show a normal organization (tj). Bar (**a**) = 2 μm, (**b**) = 300 nm.

The normal human BLB consists of vascular endothelial cells (VECs) surrounded by a perivascular basement membrane with pericyte processes embedded within the basement membrane that surrounds the VECs (Fig. 1a). Figure 1a shows a cross section of a blood vessel (low magnification) located in the stroma underneath the utricular macula sensory epithelia from a 62-year-old individual with normal vestibular or auditory function. The capillaries of the vestibular stroma are continuous, and the endothelial lining is without fenestrations. Higher magnification view of the normal human BLB (Fig. 1b) shows the VECs connected by tight junctions. The capillary endothelial surface facing the lumen is smooth and uniform, and without many pseudopodic extensions; the VECs contain organelles, with numerous mitochondria and a paucity of endocytic vesicles or vacuoles. There are only a few endocytic vesicles and no vacuoles within the cytoplasm of VECs (Fig. 1b). Another component of the BLB, the perivascular basement membrane, is a uniform structure with a homogeneous matrix (Fig. 1a and b). The perivascular basement membrane has an even thickness and intermingles uniformly within the abluminal portion of the VECs and pericytes (Fig. 1b). It completely surrounds the VECs and pericyte dendritic processes, and there is no rarefaction or duplication of the basement membrane. Pericytes have dendritic processes that surround VECs (not seen in this micrograph). There is a paucity of vacuoles or vesicles within the VEC or the pericyte.

### The capillary ultrastructure in a normal aging utricle

**Figure 2:**
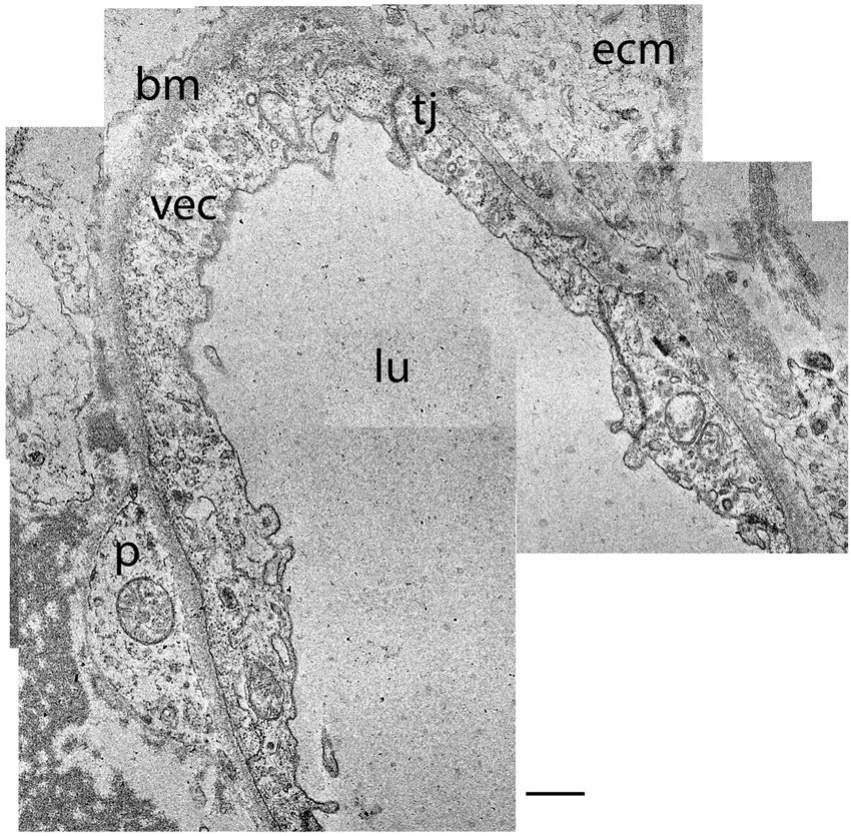
The BLB in the macula utricle from a normal subject, older age (86-year-old female). Note the normal appearance of the VECs (vec), the basement membrane (bm), extracellular matrix (ecm), tight junctions (tj), and capillary lumen (lu) are also normal. The pericyte (p) cytoplasm appears normal. Bar = 2.5 μm.

Figure 2 shows a cross section of a blood vessel (low magnification) located in the stroma underneath the utricular macula sensory epithelia from an 86-year-old individual with normal vestibular function. The VEC demonstrates few vesicles, and the tight junctions remain intact. The basement membrane is smooth, but mildly thickened in comparison with the capillary basement membrane from a younger subject. A pericyte process with normal basement membrane is easily identified.

### The BLB in subepithelial capillaries of the utricular stroma in Meniere’s disease

**Figure 3:**
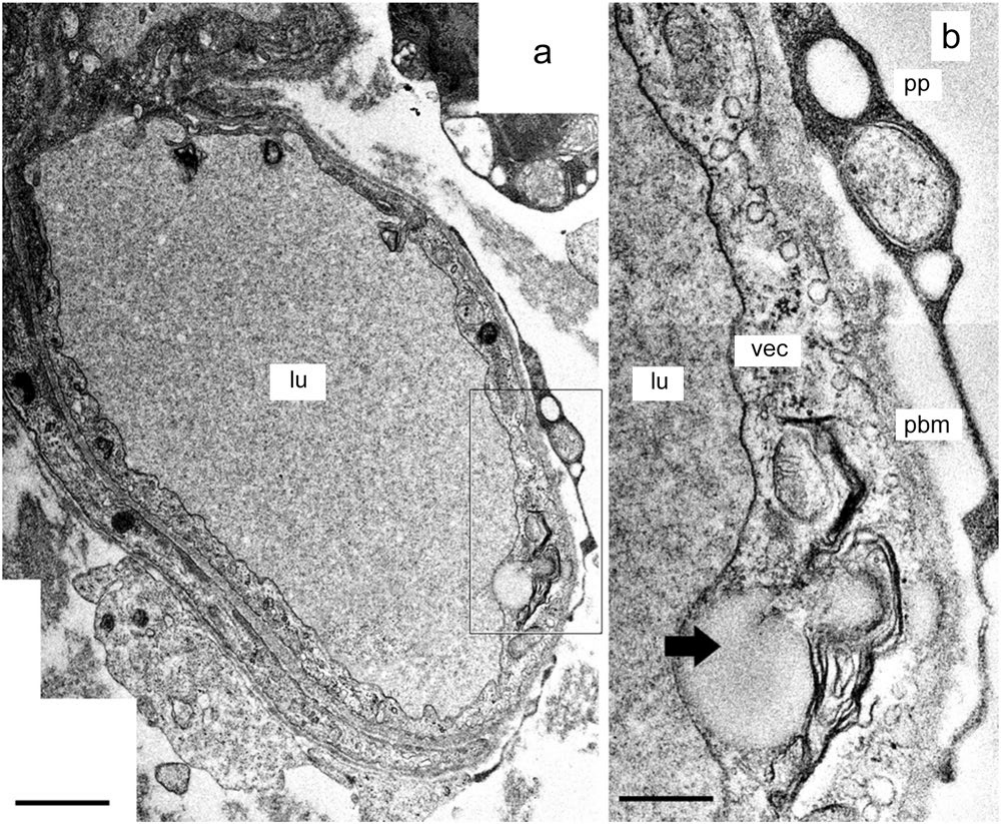
The BLB in Meniere’s disease (39-year-old). Capillary located in the utricle stroma underneath the vestibular sensory epithelia. (**a**) Low magnification view. VECs (vec) show an almost normal appearance, lumen is normal (lu), the extracellular matrix shows edema. (**b**) High magnification view -insert from (**a**). Abundant open caveolar flask-like structures that almost touch the abluminal membrane of the vascular  endothelial cell (vec) were observed. The border of the basal membrane (pbm), is almost normal, a pericyte process (pp) is detached. Thick arrowhead points to a large vacuole in the vascular endothelial cell cytoplasm. Bar in (**a**) = 5 μm, (**b**) = 2 μm.

We examined the ultrastructural changes of the capillaries located within the stroma of the macula utricle from Meniere’s disease patients. There were cases with mild or severe (ischemic) morphological alterations in the VECs. The VECs exhibited abundant vesicular transport and cytoplasmic vacuolization, pseudopodic protrusions into the lumen, microvilli-like projections, and swelling and degeneration of the organelles. In some cases, there were complete degenerative changes and necrosis of the VECs. The perivascular basement membrane, similarly, ranged from normal appearance to severely vacuolated and thickened. VECs with active vesicular transport exhibited hypertrophic Golgi complexes and markedly increased vesicle formation. Figure 3a shows a relatively intact and smooth lumen of a capillary, which demonstrates mild alterations (39-year-old with delayed endolymphatic hydrops, with history of profound hearing loss as a child with intractable vertigo spells despite medications). The VECs demonstrate some vacuoles and a prominent increase in vesicular transport facing the abluminal side, with some vesicles opening onto the abluminal area (Fig. 3b). The perivascular basement membrane is relatively intact, with uniform thickness. The VEC show mild vacuolar changes. There appears to be detachment of a pericyte process (Fig. 3b), and there are mild edematous changes within the basement membrane.

**Figure 4:**
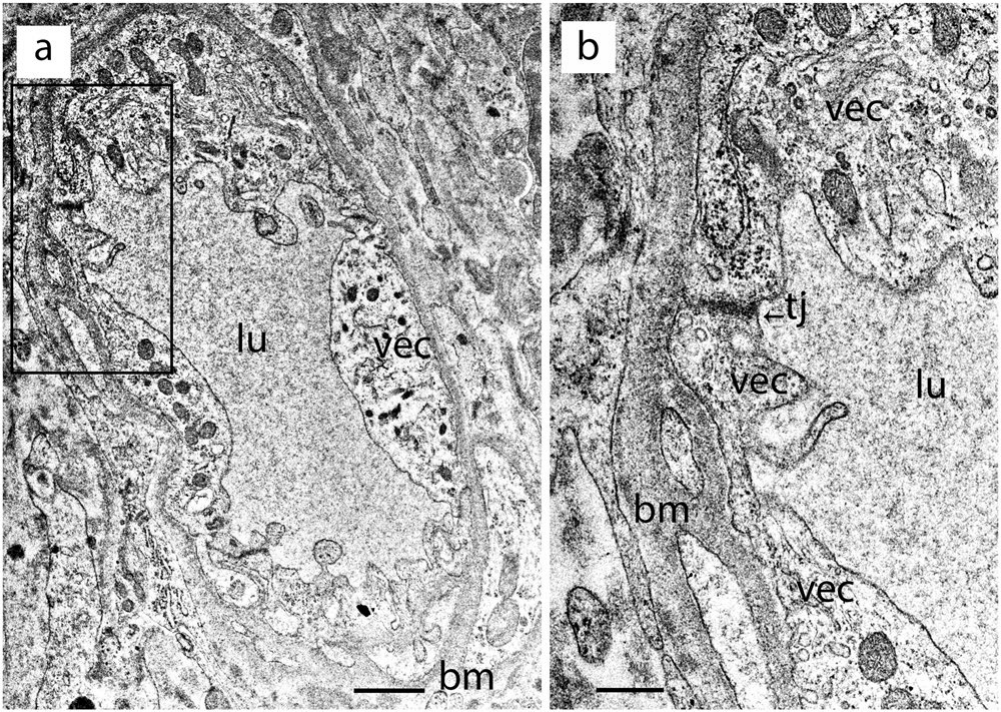
A BLB capillary in Meniere’s disease (82-year-old). Capillary located in the utricle stroma underneath the vestibular sensory epithelia. (**a**) Low magnification view. The vascular endothelial cell (vec) is swollen with degeneration of organelles and debris within the cytoplasm, the lumen (lu) is uneven and narrowed with pseudopodic and microvilli-like projections, the perivascular basement membrane (bm) exhibits mild thickening. (**b**) High magnification view (from fig a) of the vascular endothelial cell (vec). The tight junction (tj) appears normal, bm: basement membrane. Bar (**a**) = 1 μm, (**b**) = 0.5 μm.

Figure 4 shows the cross section of a capillary located in the stroma of the macula utricle from an 82-year-old male Meniere’s patient with Tumarkin falls and progressive severe loss of caloric function. The VECs exhibit swelling and numerous cytoplasmic vesicles, with narrowed and uneven capillary lumen (Fig. 4a). At higher magnification, vesicles in the endothelial cell cytoplasm are lined up polarized to the abluminal membrane. There are chained or coalesced pinocytotic vesicles forming tubular channel-like formations. Tight junctions joining VECs appear to be intact with no ultrastructural changes apparent. The perivascular basement membrane is mildly disorganized, and the extracellular matrix demonstrates mild edema. The endothelial cell lumen demonstrates multiple areas of microvilli- like projections into the lumen, and protrusion of pseudopodic expansion of the endothelial cell. There is abundant abluminal oriented vesicular transport, facing the basement membrane with some vesicles opening onto the basement membrane. Other areas of the lumen epithelium demonstrate deep invaginations that can be seen ending in micropinocytic vesicles. At higher magnification (Fig. 4b) apparent activation of a Golgi complex within the VEC can be seen.

**Figure 5:**
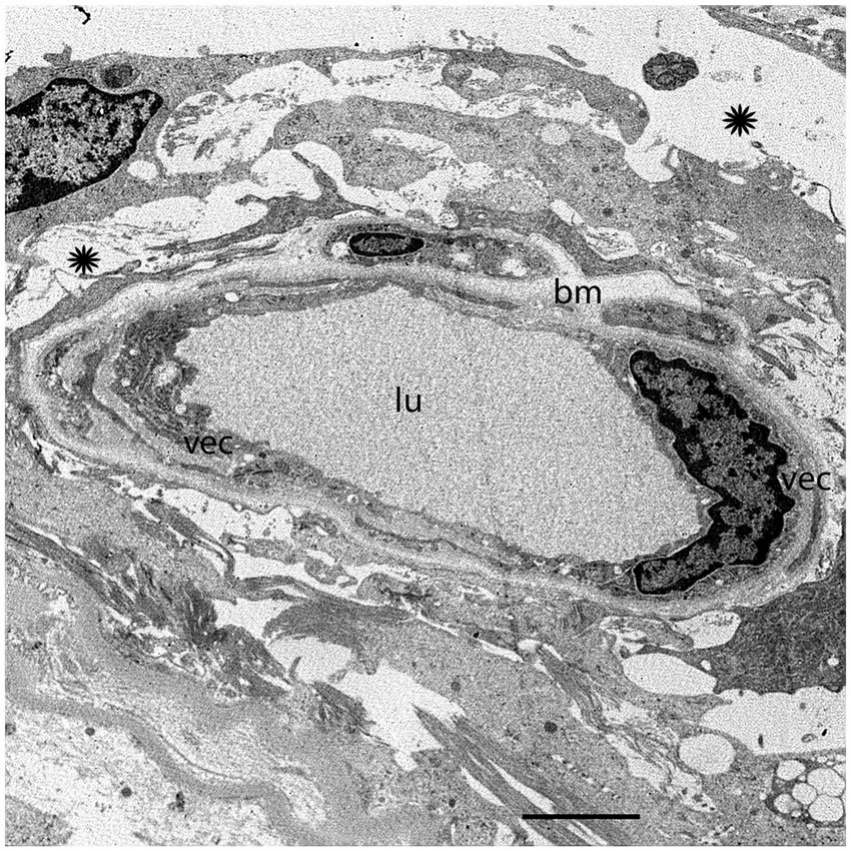
A BLB capillary with moderate degenerative changes in Meniere’s disease (40-year-old). Vacuolization is seen in vascular endothelial cells (vec), and there is apparent degeneration with loss of organelles and thinning. The perivascular basement membrane (bm) shows thickening. Lumen (lu). The stroma shows signs of edema (asterisk). Bar = 2 μm.

**Figure 6:**
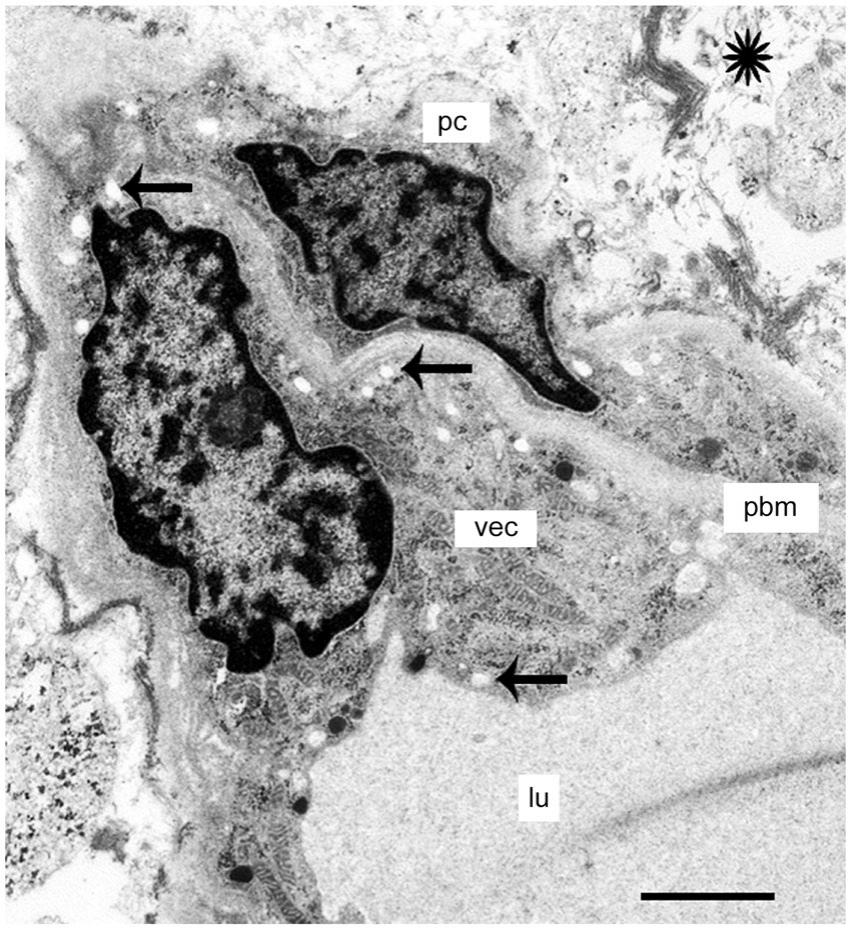
A BLB capillary with moderate degenerative changes in Meniere’s disease (56-year-old). A capillary shows moderate vacuolization of the vascular endothelial cell (vec) and pericytes (pc) and mild edema in the stroma (arrows). The perivascular basement membrane (pbm) is thickened. The stroma shows signs of edema (asterisk). Bar = 2 μm.

**Figure 7:**
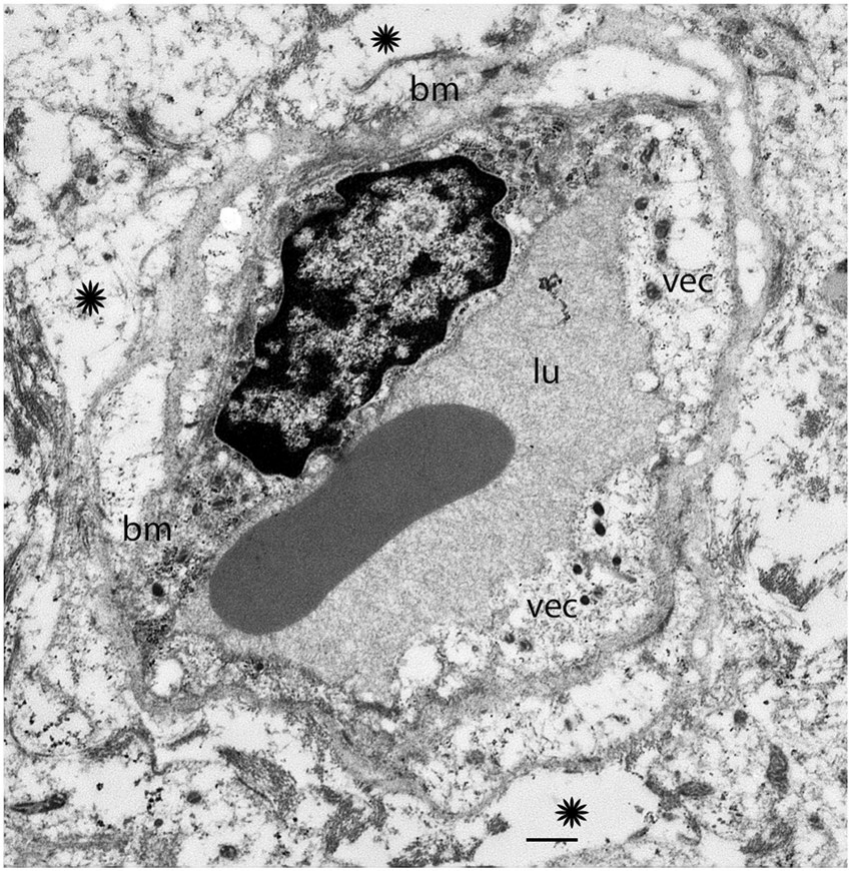
Ultrastructure of a BLB capillary with severe degenerative changes in Meniere’s disease (67-year-old). A capillary located in the macula utricle stroma. The vascular endothelial cell (vec) exhibits degenerative changes with no subcellular organelles and edematous change. The perivascular basement membrane (bm) is disorganized and swollen. There is constriction of the lumen (lu) and edema (asterisk). Bar = 2 μm.

Figure 5 shows the cross section of a capillary located in the stroma of the macula utricle from a 40-year-old individual with congenital deafness, intractable vertigo spells associated with unilateral aural fullness, and caloric paresis. The VEC is degenerated, with loss of organelles, thinning and extensive vacuolization. There is vacuolization of the extracellular space. The basement membrane is thickened, edematous, and irregular. Figure 6 shows the cross section of a capillary located in the stroma of the macula utricle from a 56-year-old with Meniere’s disease with a history of aural fullness, tinnitus, deafness and intractable vertigo spells. The putative endothelial cell demonstrates diffuse vacuolization and loss of organelles. The perivascular basement membrane is thickened and irregular, and the vacuolizations appear to be increased in the boundary. The extracellular perivascular space exhibits vacuolizations and edema. Figure 7 shows the cross section of a capillary located in the stroma of the macula utricle from a 67-year-old individual with Meniere’s disease characterized by intractable vertigo spells, Tumarkin falls, and unilateral deafness. There is severe perivascular basement membrane disorganization, and diffuse edema in the extracellular matrix. The VECs are severely swollen, with apparent necrosis and no identifiable subcellular organelles.

## Discussion

The stroma in the normal utricle is rich in capillaries, with less abundant capillaries in the deeper portion of the sensory endorgan areas, and an abundance of capillaries in the subepithelial region. There is a particularly rich network of fine capillaries beneath the basement membrane of the sensory areas. In the capillaries of the human vestibular stroma, the VECs are thin walled (2–4 μm) with a paucity of pinocytotic vesicles, and pericytes engulf the VECs via dendritic processes. In Iurato *et al*.^27^ study of the capillaries of the mammalian crista ampullaris, the flat endothelial cells are noted to be about 0.1 to 1.4 μm, with the outer surface of the VEC in close contact with the basement membrane, and the inner surface facing the capillary lumen. There are no fenestrations in the VECs in the normal rodent cristae ampullaris with cell junctions forming zonulae adherents. Similarly, in this study we found that the capillaries of the human vestibular utricular maculae are of continuous type, and the intercellular junctions between VECs are separated by tight junction-like structures. The morphological organization resembles that found in the inner ear of several animal models^28^. The basal lamina of the capillaries in the human utricular stroma is composed of type IV collagen, laminin and other proteins^29^, interposed between VECs and pericytes processes.

The site of perilymph production remains controversial, in general it is believed that it is formed as an ultrafiltrate of blood^1, 2^ with some contribution from filtration from cerebrospinal fluid (CSF). The CSF may also filtrate via the vestibular aqueduct or perivascular channels into the perilymph. The differing protein and amino acid levels of the perilymph, blood serum, and CSF are indicative of the maintenance of ionic and protein composition as part of the function of the BLB. Blood vessels, which exhibit cationic polyethyleneimine distribution, which identifies anionic sites, following intravenous administration, are indicative of macromolecular transport systems of the BLB. In the cochlea, anionic sites are present in the basement membrane of Reissner's membrane. In the vestibule, they are localized in the basement membrane of the capillary wall, and the sensory epithelium^30^. The premature rat does not yet have fully developed anionic sites within the crista ampullaris^13^. Like the blood brain barrier (BBB), the BLB develops in the early postnatal period of the immature rat. The timing of the development of the BLB has important implications for ototoxicity of agents delivered during prenatal or postnatal periods and to premature infants.

Intracerebral cortical vessels contain a mean of five caveolae per μm^2^ in arteriolar endothelium^31^, much less than that in non-neural vessels, which likely corresponds with limited transcellular traffic of solutes in BBB capillaries. Caveolae can undergo budding and fission from the plasma membrane, and translocate to the other side. In the case of Meniere’s disease, there appeared to be increased vesicular formations, with localization of vesicles to the transluminal side. The increased vesicle formation and caveolae-like formations in the VEC, with polarization to the abluminal side, which would lead one to conclude that there is increased transcytosis, possibly vesicles being used to traffic cargo across the VECs. During BBB breakdown, there is also an increase in trans-endothelial channels in VECs^32^.

In the specimens from Meniere’s disease in the present study, the tight junctions appeared to be normal, even at high magnification microscopy and also even in severely edematous BLB cytoarchitecture. In contrast, BBB in vasogenic edema appears to be associated with altered tight junction proteins, and separation noted at tight junctions; however, it is noted that these changes occur late in the course of brain injury^32^. It is possible that there is altered expression of tight junction proteins in the VEC of Meniere’s disease, without structural changes that can be visualized. Tight junctions are not only important for the regulation of paracellular permeability, but also may be involved in cytoskeletal dynamics and cellular signaling^33^.

The tight junctions in normal human BLB are continuous. No apparent abnormalities of the tight junction were found in the BLB of Meniere’s disease specimens. All of the capillaries constituting the BLB of the normal human vestibular stroma lacked fenestrations and tight junctions were noted in the intercellular space joining two-endothelial cells, forming a tight barrier which is in alignment with freeze-fracture studies of the guinea pig blood-perilymphatic barrier^34^. Of note, the stria vascularis exhibits vastly differing permeability as well as degree of pinocytosis. Therefore, the vestibular stromal BLB is similar to that of the cochlear modiolus or spiral limbus, exhibiting a similar morphology to that of the BBB.

Through imaging studies using intravenous gadolinium, there is gathering evidence for blood labyrinthine barrier compromise in Meniere’s disease. Intravenous gadolinium enhanced magnetic resonance imaging (MRI) can be used to visualize and quantify endolymphatic hydrops^22, 35^. Gadolinium, an agent used in magnetic resonance imaging (MRI), is taken up into the perilymph, presumably via perfusion through the BLB, specifically through the blood-perilymphatic barrier. Increased gadolinium enhancement in the perilymph on the ipsilaterally inner ear affected with Meniere’s disease has been noted in multiple studies, indicative of breakdown of the BLB^36–39^. Of note, in the study of Tagaya *et al*.^37^, a high signal intensity ratio correlated with a higher grade (none, mild, or significant) of hydrops in both the cochlea and the vestibule^37^. Most recently, Pakdaman *et al*.^40^ demonstrated increased gadolinium uptake in the perilymph in Meniere’s disease ipsilateral and in the contralateral unaffected ear compared with sudden hearing loss; however, the ratio of affected to unaffected ear in Meniere’s disease was significantly greater than that of sudden hearing loss. This may be indicative that permeability alterations of the BLB may be one of the primary causes of endolymphatic hydrops or Meniere’s disease.

Intratympanic injection of lipopolysaccharide (LPS), a substance that breaks down the BLB and also the BBB, is associated with increased gadolinium perilymphatic enhancement^41^. A similar increase in gadolinium uptake in the perilymph has been noted following high intensity impulse noise acoustic trauma^42^. The role of infiltration of inflammatory cells is controversial. LeFloc’h *et al*.^41^ showed that the reduction of ultrasmall superparamagnetic iron oxide particles, corresponded with cochlear inflammation, which was hypothesized to be secondary to macrophage infiltration. However, conclusive identification of a macrophage infiltrate using CD68 immunohistochemistry was negative. Using *in vivo* perilymph sampling of extravasated fluorescein from the vascular component, Hirose *et al*.^43^ demonstrated higher fluorescein in the perilymph in LPS treated mice. The region of BLB increased permeability would correspond to capillary beds, which travel through perilymph, filled spaces: spiral ligament, spiral limbus, modiolus, and osseous spiral lamina, and the subepithelial space within the vestibular stroma. In both, the uptake of gadolinium or fluorescein is not significant or nonexistent within the endolymph, indicative that the BLB lining the endolymphatic space is more restrictive than that lining the perilymphatic space. The studies together provide strong animal model evidence that increased gadolinium uptake within the perilymph is indicative of BLB permeability increase.

The role of cochlear macrophages is controversial, as elimination of macrophages did not alter the effect of LPS to induce vascular permeability^44^. In contrast to the cochlea, the vestibular periphery of the C57BL/6J mouse, exhibited LPS induced activation of perivascular macrophage-like melanocytes, identified using F4/80 staining, and increased vascular permeability as reflected by fluorescein isothiocyanate infiltration into the vestibular periphery^11^. The role of macrophages or perivascular macrophage-like melanocytes in the vascular permeability seen in the vestibular stromal capillaries from Meniere’s patients is unknown. However, of note, on light microscopy, there was no evidence for an infiltrate of inflammatory cells.

In nearly all Meniere’s cases evaluated, intact VECs of the vestibular BLB exhibited increased vesicular formation, polarized to the abluminal surface, with edema and vacuolization of the perivascular basement membrane and of the extracellular matrix. Similar patterns of endothelial cell activation are noted in cerebral edema, which is also associated with increased permeability of the BBB^45^. There are two main permeability barriers: the basement membrane and the endothelial cell. Increased endothelial cell vesiculation is reported in hypoxic-ischemic stroke, hypertensive encephalopathy, and brain trauma. It is important to note that our histopathological studies are limited, by necessity, to severe intractable stage IV Meniere’s disease associated with profound non-serviceable hearing. Within this group, there were varying degrees of severity of BLB damage. In some cases, the endothelial cell had undergone complete necrosis, and in other cases, active vesicular formation is noted within an endothelial cell with relative preservation of subcellular organelles such as mitochondria and endoplasmic reticulum.

The vestibular stromal BLB perivascular basement membrane exhibits thickening, edema, and duplication in Meniere’s disease inner ear. In some cases, the basement membrane appears to be swollen, and in more severe cases, exhibits rarefaction, duplication, and extreme thickening. The subepithelial basement membrane, which lies interposed between the epithelium facing the endolymph and the connective stromal tissue within the perilymph, is markedly thickened and under TEM appears to be disorganized fibrils^26^. Additionally, the composition of the subepithelial basement membrane is altered: collagen IV expression is diminished^46^, and the sub-epithelial basement membrane thickening correlated with neuroepithelial damage and vestibular hair cell loss^26^. In Meniere’s disease, both the sub-epithelial basement membrane and the capillary perivascular basement membrane forming the blood-perilymph barrier, exhibit thickening, edema, and disorganization in comparison with the normal basement membrane which is homogeneous, thin, with a continuous appearance due to a condensed filamentous pattern and homogeneous matrix.

It has been suggested that Meniere’s disease may be caused by dysfunctional inner ear blood flow^47, 48^ exacerbated by the pathological increase in vascular permeability of the blood-labyrinthine barrier^3, 49^. In severe cases, the capillary lumen is severely compromised due to edematous changes in the endothelial cells, and in some cases, due to apparent debris from necrotic endothelial and pericyte cells. These capillaries are likely to be dysfunctional to deliver nutrients to the neuroepithelium. The endothelial cell appears to be the earliest cell to exhibit damage.

The finding of increased vesicular transport in the endothelial cell of the capillaries, VEC degenerative changes, and thickening of the basement membrane of capillaries in Meniere’s disease raises the question of a possible inflammatory pathology similar to that proposed as causative in autoimmune inner ear disease. Trune and Nguyen-Huynh^50^ reviewed the cascade of events that can lead to increased permeability of the BLB mediated by inflammatory cytokines and chemokines that strip off the protective glycocalyx, exposing the endothelial cell, causing a cascade of events leading to loss of BLB integrity and breakdown of stria vascularis blood vessels. While there are no ultrastructural studies of the BLB in human autoimmune inner ear disease, a human temporal bone study of Sjogren's disease, an autoimmune disease associated with hearing loss in 25% of patients, demonstrated thickening of the stria vascularis basement membrane and an associated immunoglobulin deposition in Sjogren's patients with hearing loss^51^. There is only one report on the ultrastructure of the strial cells in the MRL-Fas/lpr mouse model of autoimmune disease^52^, capillary ultrastructure was found to be normal i.e. revealed no alterations in tight junctions of VECs or alterations in the basement membrane, however, there were signs of hydropic generation in cells surrounding the strial capillaries. In contrast, in the C3H/lpr autoimmune mouse, extensive leakage of ferritin into the perivascular tissues was noted, and a corresponding thickening of the basement membrane of the stria capillaries^53^. Other studies, however, are indicative that thickening of the basement membrane of the capillaries is non-specific, and has been noted in the aging mouse stria vascularis capillary basement membrane as well^54^. In no previous study in human or mouse, however, there are adequate evaluation of the VECs in the BLB of the peripheral vestibular system to clearly document whether or not there is increase in vesicles or breakdown of tight junctions or other changes. In a recent MRI study, it was noted that the permeability of the capillaries in the inner ear of Meniere’s disease patients was much significantly greater than that in sudden sensorineural hearing loss, indicative that this increased permeability likely plays a role in the pathophysiology of Meniere’s disease^55^. The relation between inner ear autoimmune disease and Meniere’s disease remains to be identified.

**Figure 8:**
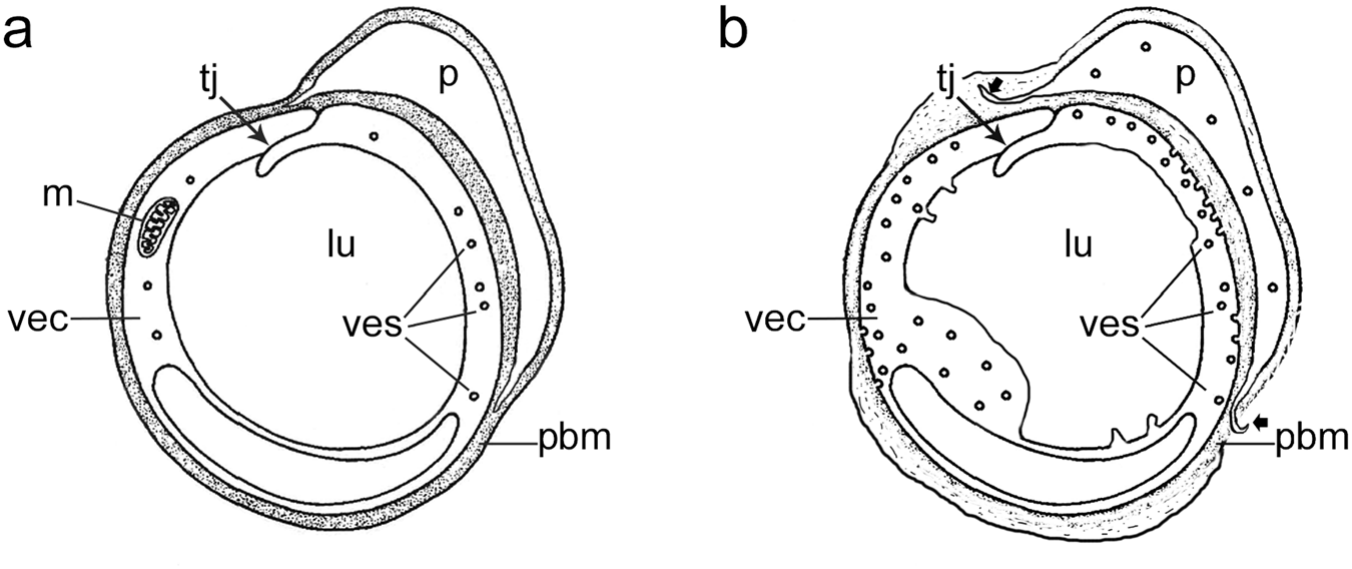
Diagram to represent the BLB in (**a**) normal capillary and (**b**) Meniere’s disease capillary. In Meniere’s disease, there are excessive vesicular formation (ves), abluminally concentrated, with degenerative changes noted early on in the endothelial cell (vec). The perivascular basement membrane (pbm) is thickened and edematous. Tight junctions (tj) are relatively preserved. Pericytes (p) exhibit vacuolization, and pericyte process detachment.

The diagram in Fig. 8a illustrates schematically the normal BLB. The BLB is maintained by tight junctions between vascular endothelial cells which form a continuous barrier without fenestrations, and are covered by a basement membrane with pericytes intermingled within the basement membrane. Diagram in Fig. 8b, shows the pathological changes noted in Meniere’s disease inner ear BLB: vascular endothelial cells exhibit edema, with an increase in vesicles, with apparent transcytosis of macromolecules. There is vacuolization of endothelial cells and pericytes, and the perivascular basement membrane exhibits thickening and duplication. In some cases, the pericyte processes are detached, and the capillary lumen is compromised by edematous changes of the endothelial cells.

In conclusion, the BLB dysfunction in the microvasculature of vestibular endorgans obtained from Meniere’s disease patients likely contributes to edematous changes in the underlying stroma and vacuolization. We hypothesize that alterations in BLB could lead to secondary changes in the vestibular sensory epithelia.

## Materials and Methods

### Specimens

The Institutional Review Board (IRB) of UCLA approved this study (IRB protocol # 10-001449). All methods used in this study were in accordance with NIH and IRB guidelines and regulations. Appropriate informed consent was obtained from each patient before inclusion in the study. Archival temporal bones were used in the present study. The temporal bone donors were part of a National Institute of Health (NIH) funded Human Temporal Bone Consortium for Research Resource Enhancement through the National Institute on Deafness and Other Communication Disorders (NIDCD). The medical history for each of the patients who had donated their temporal bones was maintained and preserved in a secured electronic database. Two maculae utricle from 2 individuals with no vestibular or auditory disease were used (62-year-old male, and 82-year-old female). Five maculae utricle from 5 patients with intractable vertigo spells or Tumarkin falls (vestibular induced involuntary drop attacks) in Meniere’s disease were studied (Table 1).

**Table 1:** Specimens (utricle) used in the present study.

Specimen	Type of Tissue	Age (years)	Gender	Diagnosis	Figure
1	Autopsy	62	Male	Normal	1a,b
2	Autopsy	86	Female	Normal	2
3	Surgical	39	Male	Meniere’s	3a,b
4	Surgical	82	Male	Meniere’s	4a,b
5	Surgical	40	Female	Meniere’s	5
6	Surgical	56	Male	Meniere’s	6
7	Surgical	67	Female	Meniere’s	7

Autopsy: Temporal bones collected between 6 to 8 hrs. postmortem, Normal: indicates normal vestibular and auditory function.

### Staging of Meniere’s disease

All subjects with Meniere’s disease had stage IV definite Meniere’s disease as defined by the American Academy of Otolaryngology- Head and Neck Surgery criteria^56^ with profound hearing loss and intractable recurrent vertigo spells despite maximum medical treatment. Patients who had previously undergone intratympanic gentamicin or endolymphatic shunt surgery were excluded.

### Transmission electron Microscopy

For ultrastructural studies, the specimens are immersed in the following solutions: 2% OsO4 and 2% potassium ferricyanide (EMS, Fort Washington, PA), 0.1% thiocarbohydrazide for 1 hr, 2% OsO4 for 30 minutes, uranyl acetate 1% overnight, and 0.1% lead aspartate for 30 minutes (5 × 3 min washes with double distilled water are made between steps). Tissue was dehydrated in ascending ethyl alcohols and embedded in resin (Epon®, EMS). Half-micrometer-thin serial sections are made with a diamond knife (Diatome) on an AO/Reichter Ultracut-E ultramicrotome. When the area of interest was visible, ultrathin 100 nm serial sections were made, and mounted on 200 mesh formvar and carbon coated copper grids (Polysciences).

### TEM image acquisition

TEM observations and digital image capture were made using a FEI Tecnai transmission electron microscope T20 TEM −200 KV (Hillsboro, Oregon USA). Morphological analysis was made in ultrathin sections containing blood vessels through the stroma of the maculae utricle. All sections are systematically analyzed at low (x 3,500-5000) and higher magnification (x 19,000-25,000). All sections were studied for the presence of vesicles and tight junctions in the endothelial cells, pericyte cytoplasmic organization, and perivascular basement membrane alterations (thickening and disruption).
